# Analysis of wheat microspore embryogenesis induction by transcriptome and small RNA sequencing using the highly responsive cultivar “Svilena”

**DOI:** 10.1186/s12870-016-0782-8

**Published:** 2016-04-21

**Authors:** Felix Seifert, Sandra Bössow, Jochen Kumlehn, Heike Gnad, Stefan Scholten

**Affiliations:** Developmental Biology, Biocenter Klein Flottbek, University of Hamburg, Ohnhorststrasse 18, 22609 Hamburg, Germany; Saaten-Union Biotec GmbH, Am Schwabenplan 6, 06466 Seeland, OT Gatersleben Germany; Plant Reproductive Biology, Leibnitz Institute of Plant Genetics and Crop Plant Research (IPK), 06466 Seeland, OT Gatersleben Germany; Institute for Plant Breeding, Seed Science and Population Genetics, University of Hohenheim, 70599 Stuttgart, Germany

**Keywords:** Microspore embryogenesis induction, Transcriptome, Small RNA, RNA-seq, sRNA-seq, Epigenetics, Wheat

## Abstract

**Background:**

Microspore embryogenesis describes a stress-induced reprogramming of immature male plant gametophytes to develop into embryo-like structures, which can be regenerated into doubled haploid plants after whole genome reduplication. This mechanism is of high interest for both research as well as plant breeding. The objective of this study was to characterize transcriptional changes and regulatory relationships in early stages of cold stress-induced wheat microspore embryogenesis by transcriptome and small RNA sequencing using a highly responsive cultivar.

**Results:**

Transcriptome and small RNA sequencing was performed in a staged time-course to analyze wheat microspore embryogenesis induction. The analyzed stages were freshly harvested, untreated uninucleate microspores and the two following stages from *in vitro* anther culture: directly after induction by cold-stress treatment and microspores undergoing the first nuclear divisions. A *de novo* transcriptome assembly resulted in 29,388 contigs distributing to 20,224 putative transcripts of which 9,305 are not covered by public wheat cDNAs. Differentially expressed transcripts and small RNAs were identified for the stage transitions highlighting various processes as well as specific genes to be involved in microspore embryogenesis induction.

**Conclusion:**

This study establishes a comprehensive functional genomics resource for wheat microspore embryogenesis induction and initial understanding of molecular mechanisms involved. A large set of putative transcripts presumably specific for microspore embryogenesis induction as well as contributing processes and specific genes were identified. The results allow for a first insight in regulatory roles of small RNAs in the reprogramming of microspores towards an embryogenic cell fate.

**Electronic supplementary material:**

The online version of this article (doi:10.1186/s12870-016-0782-8) contains supplementary material, which is available to authorized users.

## Background

Microspore embryogenesis or androgenesis involves the competence of the immature male gametophyte to switch from gametophytic to embryonic developmental cell fate through an inductive treatment prior to or at the initiation of anther or microspore culture [1]. It is an illustrative example and model for developmental plasticity and cell fate decisions in plants and an important tool in research and plant breeding for the generation of doubled haploid plants [2]. Double haploid technology is widely employed in breeding programs of many crop species for its possibility to quickly generate diverse recombinant, yet genetically fixed individuals [3]. While bread wheat (*Triticum aestivum*) is one of the globally most important crops that amount for 20 % of the human calorie consumption [4], most of its cultivars are highly recalcitrant to microspore embryogenesis. Functional genetic studies to dissect tissue culture responses are first steps in overcoming these limitations to enhance bread wheat breeding eventually. Numerous microarray based gene expression studies were conducted to elucidate the major switches from gametophytic to embryonic development in various plants [5, 6]. These experiments revealed large scale patterns in the reprogramming of microspores to embryogenic tissues, which indicated a reset of the transcriptional and translational profiles to arrest gametophytic development [7]. Nevertheless, those studies were limited by the particular microarray platform used, which likely did not cover all genes specifically expressed in the reprogramming process of microspore embryogenesis, due to a biased microarray design to transcripts expressed primarily in vegetative tissues. The advent of high throughput transcriptome analysis allows for an unlimited global analysis of expressed transcripts. Thus we performed a transcriptome sequencing (RNA-seq) study analyzing three early stages around microspore embryogenesis induction, to elucidate transcriptomic changes of two major transitions of embryogenesis induction leading to first nuclear divisions. Recently, epigenetic mechanisms were proposed to regulate the transition from gametophytic to embryogenic cell fate [8–10]. Small non-conding RNAs (sRNAs) were shown to be involved in the remodulation of the epigenetic landscape and transcript levels through different mechanisms [11], and thus are putatively potent regulators. We performed sRNA sequencing (sRNA-seq) of the same time-series as for RNA-seq, to allow for a comprehensive analysis of both sRNA and transcriptome expression changes and for the discovery of putative regulatory relationships. Our study provides the first deep sequencing-based resource for functional genomics research of microspore embryogenesis induction in wheat.

## Results and discussion

### Development of microspores and sampling

**Fig. 1:**
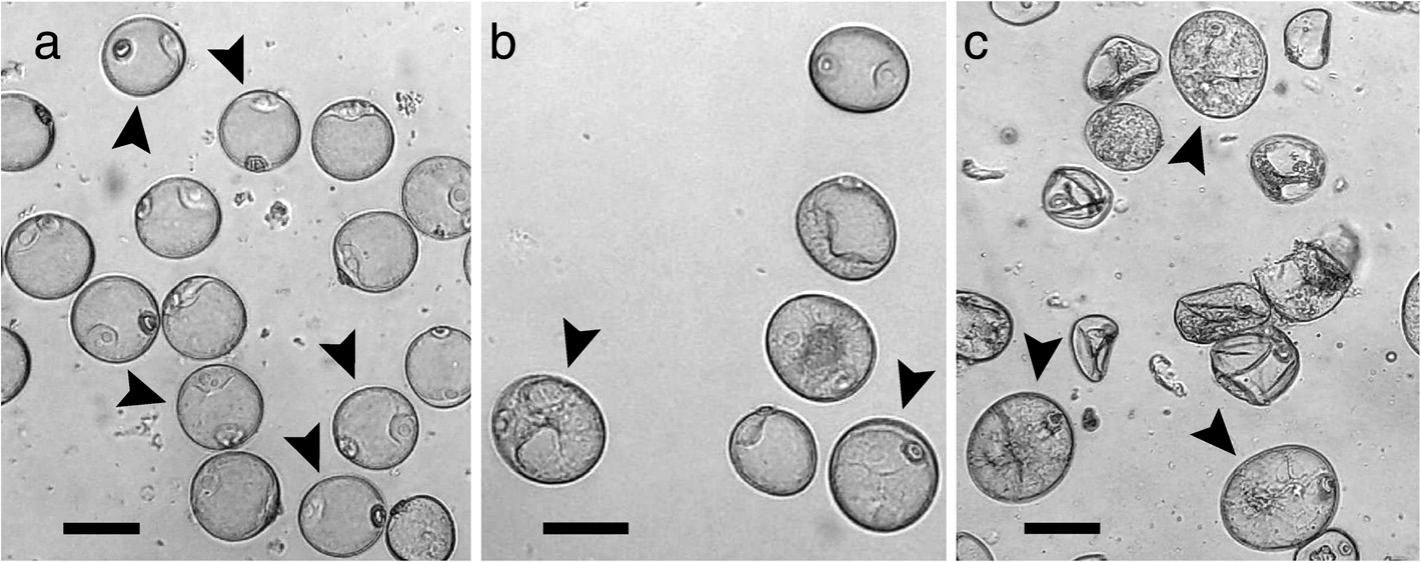
Microspore development stages sampled for RNA sequencing analysis. Brightfield micrographs of representative samples from three microspore stages. All bars represent 20 μm. Arrowheads point to cells with morphological characteristics that meet our criteria for manual cell selection. **a** Untreated vacuolated microspores at uninucleate stage (S1); manually selected microspores were characterized by a large central vacuole and a clear cytoplasm. **b** Microspores with star-like structure after 10-days cold stress pre-treatment (S2); microspores are slightly enlarged after stress induction, the vegetative nucleus migrates into the center of the cell, the cytoplasm becomes structured and shows cytoplasmic strands, the so-called “star-like structure”. **c** Microspores undergoing first nuclear division (S3); the vegetative nucleus is centrally located and has divided

Donor plants of the winter wheat cultivar “Svilena”, which is highly responsive to stress-induced microspore embryogenesis [12], were used for anther culture as described by Rubtsova et al. (2013) [13]. Microspores were sampled at three stages: a) freshly harvested microspores at their late, uninucleate highly vacuolated stage (S1), b) microspores after 10 days of cold pre-treatment exhibiting a star-like structure (S2) and c) microspores undergoing early nuclear division (S3) based on visual assessment (Fig. 1). These visually distinct developmental phases in microspore embryogenesis induction represent crucial stages in the acquisition of embryogenic potential, which were elucidated in various cytological studies [1, 7, 14]. It has been shown that microspores, before, or immature pollen, directly after pollen mitosis I, are most responsive for stress treatment-induced embryogenic development. The first effect after stress treatment is a rearrangement of the cytoskeleton resulting in the re-localisation of the nucleus to the center of the cell. The nucleus is surrounded by cytoplasmic strands and thus a star-like structure is formed by this process, which was suggested to be the first sign of embryogenic induction [2, 15]. The manual sorting procedure that we applied for RNA-seq facilitates a very high homogeneity and thus a stage-specific analysis of the pooled microspores as well as an exclusion of injured or dead cells. Due to higher RNA amount requirements for sRNA-seq, a gradient centrifugation-based isolation was performed, which delivers the required cell numbers at the cost of slightly reduced population homogeneity. To control for batch-to-batch variations, donor material for all microspore isolations for RNA-seq and sRNA-seq were cultured until plant regeneration. In either case the high regeneration frequency was equivalent to the usually observed response for the cultivar “Svilena”.

### Transcriptome sequencing

RNA-seq allows an unrestricted and global analysis of gene expression as well as the identification of unknown transcripts. To facilitate a comprehensive overview of gene expression through acquisition of embryogenic potential of microspores, we sequenced the samples in biological triplicates for each stage. All libraries were indexed with unique nucleic acid identifiers and 50 bp single end reads were sequenced on an Illumina HiSeq 2000 sequencer. In total, 608,233,335 clean RNA-seq reads were generated, with individual libraries covering 55.6 Mio. to 75.6 Mio. reads (see Table 1).

**Table 1:** Summary of RNA-seq/sRNA-seq data

	RNA-seq data		sRNA-seq data (18 to 28-nt)	
Sample replicate	Trimmed reads	Uniquely mapping reads to *de novo* transcriptome [%]	Trimmed reads	Distinct reads
S1a	70,216,602	39.442	10,425,301	1,813,927
S1b	75,697,405	38.307	10,247,910	2,332,967
S1c	55,646,245	34.298	9,892,496	2,983,585
S2a	66,434,325	42.742	10,707,291	3,092,207
S2b	66,122,827	43.012	9,718,833	2,572,135
S2c	73,711,917	42.240	10,154,174	3,174,185
S3a	71,321,690	44.759	10,789,357	2,908,387
S3b	70,400,647	50.624	10,664,717	3,063,376
S3c	58,681,677	40.775	9,939,297	4,006,577

### *De novo* transcriptome assembly and annotation

**Fig. 2:**
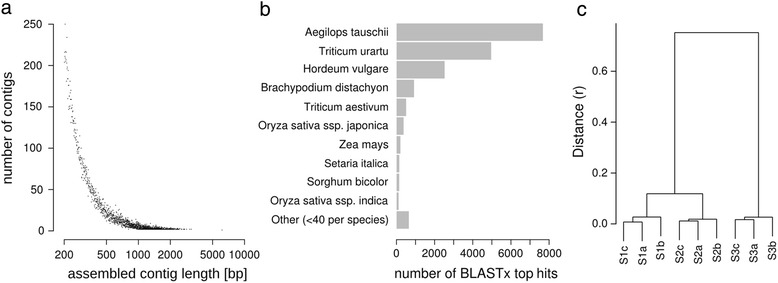
Results from RNA-seq transcriptome assembly and expression analysis. **a** Size distribution of contigs assembled from RNA-seq reads of all replicates of the three microspore stages using the Trinity assembler. **b** Species distribution for BLASTx top hits of RNA-seq assembled contigs against the NCBI nr database. **c** Correlation-based clustering analysis for RNA-seq transcript expression values between the replicates of all microspore stages

A *de novo* transcriptome assembly using the Trinity *de novo* assembler [16] was performed based on the RNA-seq reads of all stages and replicates and resulted in 29,388 contigs with an average length of 417.87 bp. The size distribution of the contigs is shown in Fig. 2a. Our approach allows for an expression comparison as well as a functional annotation for the identification of important gene functions in microspore embryogenesis induction. We did not pursue resolving the homeologs or isoforms, this would have required a higher sequencing depth as well as longer and paired end reads. A BLASTx mapping resulted in 18,344 (62.42 %) contigs with homology to protein sequences in the NCBI nr database. The majority of contigs exhibits the highest sequence homology with *Aegilops tauschii* and *Triticum urartu*, known to be the diploid progenitors for the wheat A and D genome, respectively [17], followed by other grass species (Fig. 2b). This indicates wheat specific sequencing results without contamination and an effective *de novo* assembly resulting in high homology to known monocot transcripts.

We annotated the contigs by assigning gene ontology (GO) terms via Blast2GO [18] and Trinotate [19]. This annotation resulted in 13,553 (46.12 %) contigs with a homology-based annotation, with on average 7.41 GO terms per contig. Mapping of the contigs to known wheat cDNA sequences (ensembl release 26) [20] resulted in 10,919 cDNAs covered by contigs from the RNA-seq *de novo* assembly. A subset of 5,139 wheat cDNAs were covered by multiple contigs (on average 2.78 contigs per cDNA) most likely due to fragmented assembly of the short reads. The restructuring of the contigs to transcripts based on wheat cDNA sequences revealed 20,224 transcripts covered by our *de novo* assembly. The contig assignment to transcripts is listed in Additional file 1: Table S1. This restructured assembly contains 9,305 new transcripts not covered by known wheat cDNAs from ensembl release 26 [20], presumably because the specific cell-types, developmental stages and induction conditions used in the present study were not covered by previous sequencing efforts. Our dataset thus provides a valuable resource for the analysis of microspore embryogenesis. 3,206 (32.21 %) of the new transcripts could be annotated by BLASTx mapping. The top hits from BLASTx for contigs attributed to restructured transcripts are shown in Additional file 2: Table S2. After the restructuring of contigs to transcripts, a GO annotation could be derived for 8,527 (42.16 %) of all transcripts including 996 transcripts not covered by wheat cDNAs (Additional file 3: Table S3). The GO annotation resulted in a large number of transcripts with biological processes related to response to stress and abiotic stimulus, which is most likely caused by the cold-stress treatment for microspore induction. Other main biological processes covered are cellular component organization, post-embryonic development, cell cycle, cell differentiation, embryo development and epigenetic regulation of gene expression (Table 2), which might be related to the developmental shift from gametophytic to embryogenic cell fate. The complete list of GO terms for all categories is shown in Additional file 4: Table S4.

**Table 2:** Number of transcripts covered by GO terms of GO category biological process (*n* > =100)

GO term	GO description	Number of transcripts
GO:0009987	cellular process	3037
GO:0009058	biosynthetic process	1457
GO:0006950	response to stress	1373
GO:0016043	cellular component organization	1364
GO:0006810	transport	1232
GO:0009056	catabolic process	1197
GO:0008152	metabolic process	1147
GO:0006139	nucleobase-containing compound metabolic process	1094
GO:0006464	cellular protein modification process	1094
GO:0009628	response to abiotic stimulus	913
GO:0005975	carbohydrate metabolic process	763
GO:0008150	biological process	757
GO:0006350	transcription, DNA-templated	701
GO:0007275	multicellular organismal development	699
GO:0019538	protein metabolic process	676
GO:0006259	DNA metabolic process	587
GO:0009791	post-embryonic development	578
GO:0000003	reproduction	541
GO:0006629	lipid metabolic process	517
GO:0007165	signal transduction	512
GO:0007049	cell cycle	498
GO:0009653	anatomical structure morphogenesis	498
GO:0009607	response to biotic stimulus	482
GO:0006412	translation	427
GO:0006519	cellular amino acid metabolic process	376
GO:0009719	response to endogenous stimulus	369
GO:0030154	cell differentiation	356
GO:0009908	flower development	329
GO:0009790	embryo development	302
GO:0006091	generation of precursor metabolites and energy	277
GO:0040029	regulation of gene expression, epigenetic	256
GO:0016049	cell growth	224
GO:0019748	secondary metabolic process	209
GO:0006355	regulation of transcription, DNA-templated	183
GO:0055114	oxidation-reduction process	180
GO:0006351	transcription, DNA-templated	170
GO:0006468	protein phosphorylation	149
GO:0006886	intracellular protein transport	118
GO:0009605	response to external stimulus	112
GO:0008219	cell death	109
GO:0055085	transmembrane transport	107
GO:0006457	protein folding	100

**Table 3:** Overrepresented biological processes of transcript expression clusters

Cluster	GO term	GO term description	Number of transcripts	Enrichment p-value
1	GO:0006259	DNA metabolic process	90	<10^-6^
1	GO:0007049	cell cycle	78	<10^-6^
1	GO:0007018	microtubule-based movement	12	3 · 10^-6^
1	GO:0007067	mitotic nuclear division	12	3 · 10^-6^
1	GO:0040029	regulation of gene expression, epigenetic	44	1.1 · 10^-5^
1	GO:0043531	ADP binding	16	1.6 · 10^-5^
1	GO:0006275	regulation of DNA replication	12	7.9 · 10^-5^
1	GO:0003677	DNA binding	111	9.6 · 10^-5^
1	GO:0004803	transposase activity	8	1.86 · 10^-4^
1	GO:0006260	DNA replication	15	2.86 · 10^-4^
1	GO:0003774	motor activity	12	3.81 · 10^-4^
1	GO:0006418	tRNA aminoacylation for protein translation	10	7.99 · 10^-4^
1	GO:0008017	microtubule binding	6	9.88 · 10^-4^
1	GO:0006313	transposition, DNA-mediated	7	1.01 · 10^-3^
1	GO:0007131	reciprocal meiotic recombination	9	1.21 · 10^-3^
1	GO:0000911	cytokinesis by cell plate formation	11	1.69 · 10^-3^
1	GO:0006281	DNA repair	18	2.16 · 10^-3^
1	GO:0010332	response to gamma radiation	7	2.95 · 10^-3^
1	GO:0006261	DNA-dependent DNA replication	7	3.88 · 10^-3^
1	GO:0009909	regulation of flower development	13	4.31 · 10^-3^
1	GO:0003676	intracellular protein transport	77	5.49 · 10^-3^
1	GO:0006886	intercellular protein transport	19	6.59 · 10^-3^
1	GO:0016043	cellular component organization	140	8.09 · 10^-3^
5	GO:0009987	cellular process	205	5.18 · 10^-4^
5	GO:0019538	protein metabolic process	59	5.25 · 10^-4^
5	GO:0051603	proteolysis involved in cellular protein catabolic process	5	1.3 · 10^-3^
5	GO:0005839	proteasome core complex	5	1.75 · 10^-3^
5	GO:0006810	transport	91	3.29 · 10^-3^
9	GO:0009220	pyrimidine ribonucleotide biosynthetic process	7	1.1 · 10^-4^
9	GO:0003735	structural constituent of ribosome	12	3.43 · 10^-4^
9	GO:0006412	translation	25	3.16 · 10^-3^
9	GO:0006094	gluconeogenesis	5	3.68 · 10^-3^
9	GO:0009560	embryo sac egg cell differentiation	5	5.98 · 10^-3^
10	GO:0009058	biosynthetic process	85	<10^-6^
10	GO:0006629	lipid metabolic process	42	<10^-6^
10	GO:0009987	cellular process	138	4.7 · 10^-5^
10	GO:0009056	catabolic process	65	1.17 · 10^-4^
10	GO:0019748	secondary metabolic process	19	1.37 · 10^-4^
10	GO:0008152	metabolic process	62	2.1 · 10^-4^
10	GO:0005975	carbohydrate metabolic process	44	5.72 · 10^-4^
10	GO:0019538	protein metabolic process	39	1.17 · 10^-3^
10	GO:0006091	generation of precursor metabolites and energy	19	3.9 · 10^-3^
10	GO:0006519	cellular amino acid metabolic process	23	6.15 · 10^-3^
11	GO:0009058	biosynthetic process	60	<10^-6^
11	GO:0008152	metabolic process	45	<10^-6^
11	GO:0006629	lipid metabolic process	35	<10^-6^
11	GO:0005975	carbohydrate metabolic process	35	<10^-6^
11	GO:0006091	generation of precursor metabolites and energy	21	<10^-6^
11	GO:0019748	secondary metabolic process	17	<10^-6^
11	GO:0009056	catabolic process	42	1.8 · 10^-5^
11	GO:0005488	binding	51	1.09 · 10^-4^
11	GO:0006096	glycolytic process	7	2 · 10^-4^
11	GO:0009987	cellular process	77	2.75 · 10^-4^
11	GO:0006519	cellular amino acid metabolic process	17	5.5 · 10^-4^
11	GO:0015979	photosynthesis	7	6.02 · 10^-4^
11	GO:0055114	oxidation-reduction process	10	1.86 · 10^-3^
11	GO:0009628	response to abiotic stimulus	29	2.28 · 10^-3^
11	GO:0006950	response to stress	39	2.8 · 10^-3^

### Expression analysis

The expression levels of all transcripts were estimated based on uniquely mapping reads to the *de novo* assembled transcriptome (see Table 1). To allow for a comparison of replicates and stages the expression values were quantile normalized and scaled to one million quantile normalized reads per library (rpmqn). Correlation based clustering revealed that the expression values between the replicates exhibited a high similarity for each of the three specific stages and a clear separation from the other two stages (Fig. 2c). This clearly indicates that the manually sorted cells represent uniform samples of developmentally distinct stages. Additionally we observed a much higher overall similarity between the transcriptomes of the stages S1 and S2 than between the first two stages and S3 (Fig. 2c). This result suggests, that the stress treatment causes few but drastic changes that direct to a large-scale reprogramming in the following transition.

**Fig. 3:**
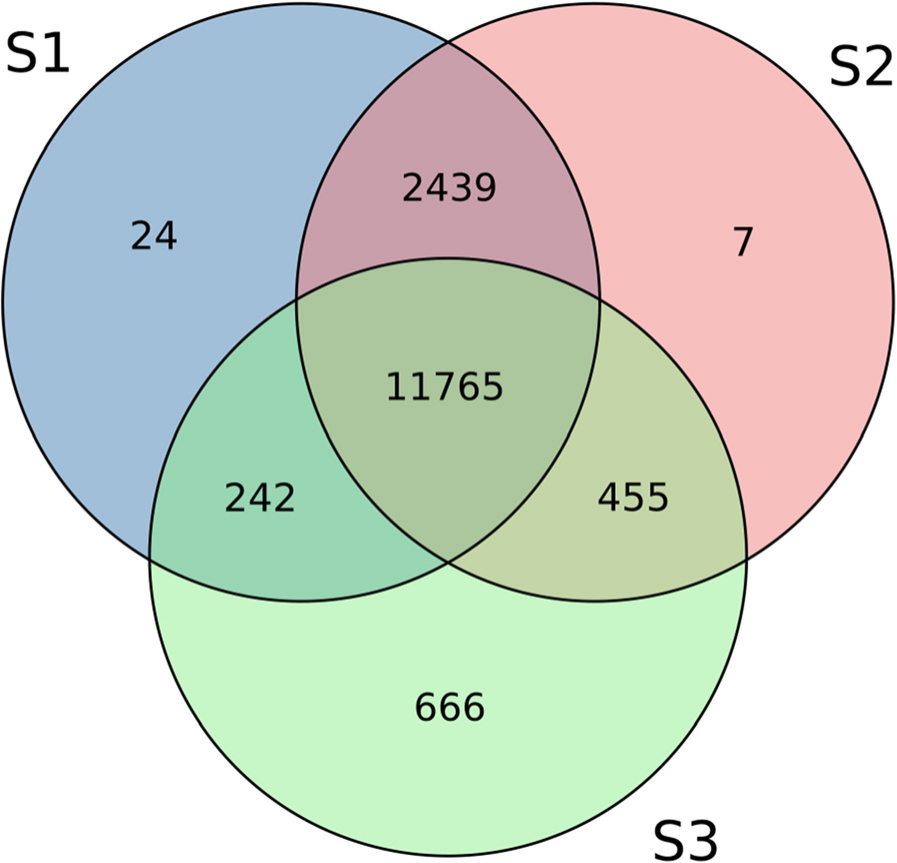
Overlap of expressed transcripts in the three analyzed stages

For the analysis of stage specific transcription, we regarded transcripts with an expression of at least 1 rpmqn in all three replicates of at least one of the three stages as expressed. These thresholds revealed 14,792 (73.14 %) transcripts to be expressed in S1, an increase to 15,026 (74.3 %) expressed transcripts in S2 followed by a decrease to 13,927 (68.86 %) expressed transcripts in S3, respectively. The overlap of transcripts exclusively expressed in the stages S1 and S2 is 2,439 (12.06 %) transcripts, but only 455 (2.25 %) transcripts were exclusively expressed in the stages S2 and S3 (see Fig. 3). A core set of 11,765 (58.17 %) transcripts was expressed in all three stages. The differing sets of expressed transcripts reflect the change of developmental fate in the transcriptome. Microspores that eventually develop into embryos have been shown to undergo a step of dedifferentiation first, which is completed at the stage exhibiting a star-like structure [7]. We found 24, 7, and 666 transcripts to be exclusively expressed in S1, S2, and S3, respectively (see Fig. 3). The transcripts along with their BLASTx top hits are listed in Additional file 5: Table S5. Interestingly, transcripts exclusively expressed in S3 cover transcripts which are known to be involved in acquisition of embryogenic cell fate, like transcript_14378 and transcript_18369 with similarity to *RWP-RK DOMAIN CONTAINING 1* (*RKD1*), a transcription factor involved in female gametogenesis and early embryogenesis identified from isolated wheat egg cells [21]. transcript_7306 with similarity to *AINTEGUMENTA-like 5* (*AIL5),* an AP2-like ethylene-responsive transcription factor, which is a homolog to *BABY BOOM* (*BBM*) and known to confer embryonic identity to cells [22]. transcript_11677 exhibits similarity to *HIGH-LEVEL EXPRESSION OF SUGAR-INDUCABLE GENE2-LIKE1* (*HSL1)*, which was shown to be specifically and highly expressed in early embryogenesis. Its interaction with the *HISTONE DEACETYLASE 19* (*HDA19*) results in epigenetic repression of seed maturation genes [23]. Another epigenetic component, exclusively expressed in S3, is transcript_12642 with similarity to *SHOOTLESS2* (*SHL2*), an orthologue of the *Arabidopsis* RNA-dependent RNA polymerase 6, which was shown to be involved in shoot apical meristem formation during embryogenesis [24]. Additionally, the specific expression of transcript_13594 and transcript_20002 in S3, both with homology to the *DNA (cytosine-5)-methyltransferase 1A* (*MET1a*), is in agreement with reported DNA methylation dynamics and *MET1a*-like gene expression changes during stress-induced microspore reprogramming [25]. Overall, the large number of transcripts with homologies to known embryogenesis related genes suggests that we have identified many more not yet uncovered genes related to wheat microspore embryogenesis induction.

### Analysis of differentially expressed transcripts

The transitions between the stages S1 and S2 (in the following denoted as T1) as well as between S2 and S3 (named T2) represent pivotal steps in induction and reprogramming from gametophytic fate of the microspore into embryo formation [7]. The differential expression (DE) of transcripts was determined for all transcripts with at least 2 reads per million quantile normalized reads (rpmqn) in the higher expressed stage, and a two-fold expression change in the transition between the respective stages. The expression analysis resulted in 756 DE transcripts for the first transition (T1) and 5,629 DE transcripts for T2 (Additional file 6: Table S6). In both transitions the majority of transcripts is downregulated, 66.67 % in T1 and 56.96 % in T2. 301 (39.81 %) of the DE transcripts after the cold-stress treatment in T1 exhibit also DE in T2. The proportion of the number of up- and downregulated transcripts in T1 resembles a previous microarray-based study for the effect of mannitol-treatment on microspore embryogenesis in barley [26].

The correlation-based cluster analysis of the expression stage specific expression values (Fig. 2c) suggested more differences in gene expression in T2 than in T1. These results were supported by a principal component analysis (PCA) for all DE transcripts in at least one stage transition, which resulted in a clear separation of the first two microspore stages S1 and S2 from the later stage S3, explaining 72.45 % of the variance (Additional file 7: Figure S1). The similarity of S1 and S2 in comparison to S3 in the PCA highlights that this separation pattern is not a result from higher expression variation between the replicates that could have been potentially caused by the manual sampling of the microspores, but differential expression of specific sets of transcripts.

**Fig. 4:**
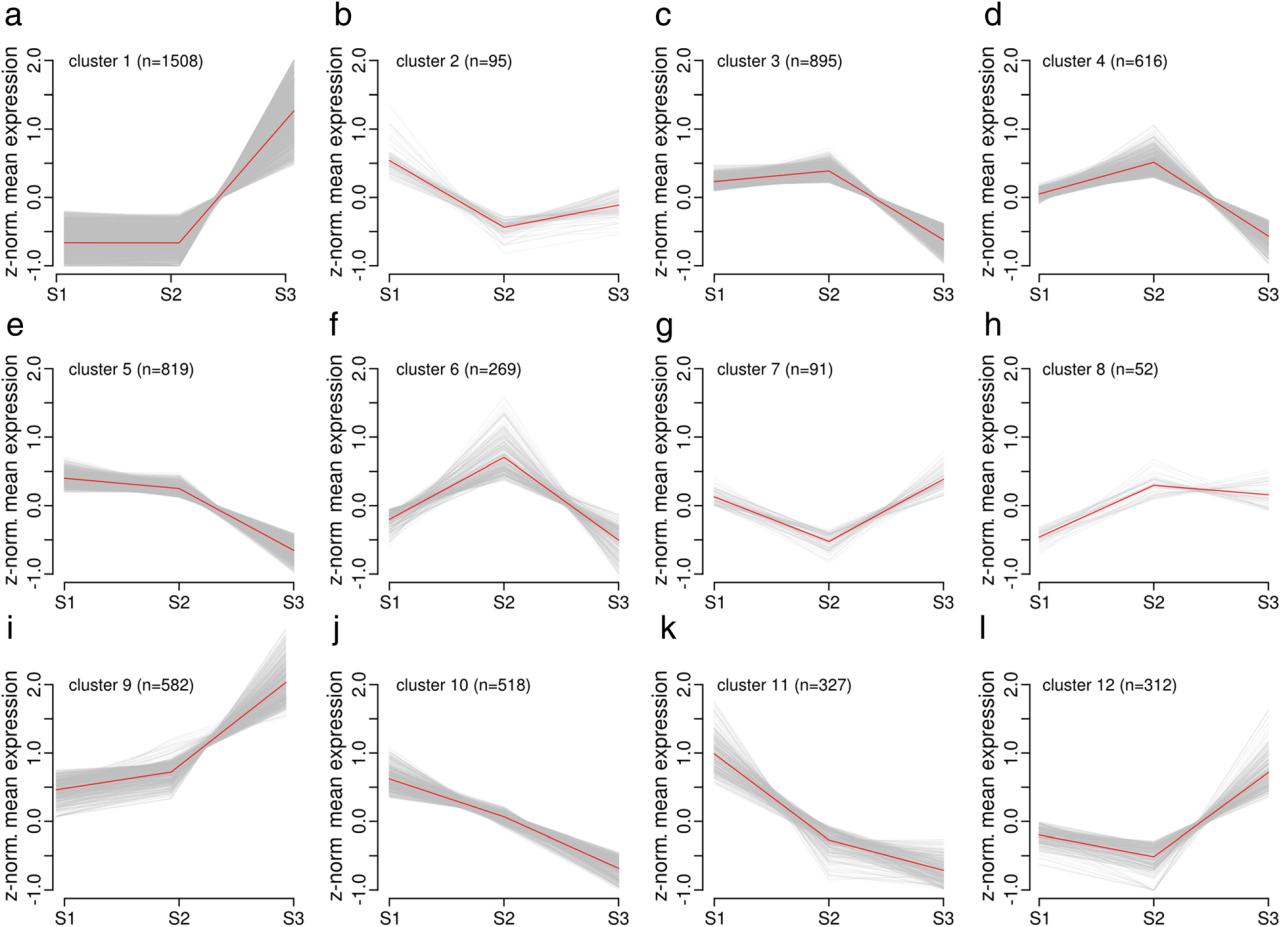
Clustering of DE transcript expression profiles. Representation of DE transcript expression profiles derived from k-means clustering of expression z-scores. The red line shows average expression z-scores to visualize the dominant expression trend of the cluster. **a** cluster 1, **b** cluster 2, **c** cluster 3, **d** cluster 4, **e** cluster 5, **f** cluster 6, **g** cluster 7, **h** cluster 8, **i** cluster 9, **j** cluster 10, **k** cluster 11, **l** cluster 12. The number of transcripts (n) is given for each cluster

A k-means cluster analysis for all DE transcripts was performed to uncover expression switches throughout the two stage transitions (see Fig. 4). In agreement with the expression comparison (Fig. 2c) as well as with the results from the PCA the clustering resulted predominantly in two major expression pattern clusters, with basically either up (cluster 1, 9 and 12; see Fig. 4a, Fig. 4i and Fig. 4l) or down (cluster 3 and 5; see Fig. 4c and Fig. 4e) regulation of expression between the microspore stages S2 and S3. Another expression pattern is up-/downregulation specifically after the stress treatment in T2 with reversion of the expression pattern towards T3 given for clusters 4, 6 and 7 (see Fig. 4d, Fig. 4f and Fig. 4g). Interestingly only clusters exhibiting a steady decrease (cluster 10 and 11; see Fig. 4j and Fig. 4k) but none for steady increase of gene expression could be observed. Changes in gene expression either up or down in T1 is given only for a smaller number of transcripts (cluster 2 and 8; see Fig. 4b and Fig. 4h).

The clusters were inspected for known regulatory transcripts, which signify the transition from the gametophytic to the embryonic developmental program. Strikingly, cluster 1 contains a transcript with homology to the embryogenesis related transcription factor *BABY BOOM 2* (*BBM2,* transcript_4758). Interestingly, the major clusters 1 and 3 both contain various transcripts with homology to epigenetic components such as the Argonaute genes *AGO4* (transcript_3992), *AGO5* (transcript_1301) and *AGO6* (transcript_2354), the dicer-like gene *DCL3* (transcript_378), a large number of chromatin remodelling factors, such as various DNA-methyltransferases (*DDM1,* transcript_1568; *DRM2,* transcript_1831; *MET1*, transcript_3460; *CMT3*, transcript_6805), histone methyltransferases (*NSD3*, transcript_633; *SUVH1*, transcript_1921; *SUVR*5, transcript_1884) as well as the histone deacetylase (*HD2A*, transcript_5605) in cluster 1. The opposing cluster 3 contains *DCL1* (transcript_3100), the DNA-methyltransferase (*DRM1*, transcript_5286), as well as various histone deacetylases (*HDA6,* transcript_4537; *HDA19*, transcript_2818). The histone deacetylases *HDA6* and *HDA19* have been shown to be suppressors of embryonic properties [27] and thus were rightly found in cluster 3. Likewise, changes in histone methylation and acetylation are associated with cell totipotency during microspore reprogramming to embryogenesis [9]. In agreement with other studies on androgenesis in various species [8–10] the large number of epigenetic components we found to be differentially expressed between the stages highlights their importance in the reprogramming of immature microspores to embryogenic cell fate.

Interestingly, homologues of previously discussed embryogenesis-marker genes are covered by the *de novo* assembled transcripts, such as *SOMATIC EMBRYOGENESIS RELATED KINASE 1 (SERK1*) [7] or *LATE EMBRYO ABUNDANT* (*LEA*) [28]. Unexpectedly, we found *SERK1* with highest expression in fresh microspores and the expression level decreases through both transitions. This is in agreement with the finding that *SERK1* was essential for male gametophyte production [29] and indicates that its expression pattern is not exclusively attributed to embryogenic reprogramming. We found *LEA* to be expressed at low levels in all three stages without any significant changes in expression levels neither after induction-treatment (T1) nor towards induced embryogenesis (T2). Thus the transcription profiles of these known embryogenesis-marker genes do not indicate their involvement in the reprogramming of wheat microspores.

### GO enrichment analysis

To further functionally characterize the stage transitions and expression clusters we performed a GO enrichment analysis for DE transcripts. The full results are listed in Additional file 8: Table S7 and Additional file 9: Table S8, for the transitions and the expression pattern clusters, respectively. Additionally, major enrichments of the expression clusters are shown in Table 3. In T1, GO terms were only found to be enriched for downregulated transcripts, namely, amongst others, “carbohydrate metabolic process”, “vacuole”, and “response to stress”, which all likely represent the dedifferentiation of the microspores due to the inductive treatment. A large set of GO terms overlaps among the downregulated transcripts in both transitions such as “generation of precursor metabolites” and “energy”, “lipid metabolic process”, “metabolic process”, “catabolic process”, “biosynthetic process”, and “response to abiotic stimulus”, which presumably represent sustained dedifferentiation from the microgametophytic pathway. The set of GO terms for upregulated transcripts in T2 contains the general terms protein binding, DNA binding and nucleic acid binding, most likely reflecting initiation of embryogenic transcription and protein machinery. Likewise, the GO terms “cell cycle”, “cellular component organization” as well as numerous microtubule and mitosis related terms, “histone H3K9 methylation”, “DNA methylation” and “histone phosphorylation” were enriched among upregulated transcripts in T2. The indicated downregulation of metabolic and biosynthetic processes in both transitions with concurrent upregulation of chromatin modifications and organization of cellular components as well as the cell cycle in T2 is in agreement with a cell cycle arrest, which was suggested to be required for the reprogramming to embryogenic fate before the cell cycle is again released [1]. The GO term “H3K9 methylation” for upregulated transcripts in T2 is in accord with the finding of increased H3K9 methylation in embryo-like structures as compared to microspores [9].

Cluster 1 exhibits an enrichment for various GO-terms reflecting karyokinesis, the microspores are undergoing in T2, such as “microtubule binding”, “cytokinesis by cell plate formation”, “DNA-dependent DNA replication” and “cytoskeleton”. The stress-induced rearrangement of the cytoskeleton followed by a symmetric division of the microspore has been described in various studies as initial steps towards microspore embryogenesis (see review [2]). Although cluster 3 is the second largest cluster, it is only enriched for the single GO-term “endoplasmic reticulum”. That there are no other terms enriched, might reflect that the downregulation of transcript expression in T2 covers a multitude of functions and processes. In contrast, cluster 5 with a similar expression pattern but continuous downregulation in T1 and T2 has an enrichment for numerous GO-terms for protein related processes, such as “protein metabolic process”, “proteolysis involved in cellular processes”, “Golgi apparatus” and “proteasome core complex”, which might reflect the previously described degradation of gametophytic cell fate-related proteins to allow for a reprogramming towards embryogenesis. Especially, the enrichment for “Golgi apparatus” might resemble findings in *Brassica napus* where autophagy and cytoplasmic cleaning by excretion was found to be unique to microspores undergoing reprogramming to an embryogenic fate: In contrast to non-responding microspores, freshly isolated microspores at the vacuolated stage, which were optimal for induction, exhibit Golgi-stacks [30]. Surprisingly, the corresponding genes show equal expression levels in untreated isolated microspores and after the stress-treatment. This might indicate an early transcriptional stress response to the mannitol buffer and would fit to a similar observation by Marashin *et al.* [3]. Cluster 9 is enriched for GO-terms related to transcription and translation: “structural constituent of ribosome”, “ribosome”, “DNA binding”, “translation” and “DNA-directed RNA polymerase activity” and is likely related to the establishment of an embryogenic program. Interestingly, cluster 9 exhibits also enrichment for the GO-term “embryo sac egg cell differentiation”, which might be indicative for the reprogramming of the microgametophytic pathway. Although cluster 10 and 11 represent progressive downregulation of transcripts and both cover only a relative small amount of DE transcripts, they exhibit enrichments for a large number of GO-terms related to catalytic activity and various metabolic processes, which might relate to downregulation of microgametophytic pathways. The additional enrichment for various stress-related terms, such as “oxidation-reduction process”, “response to abiotic stimulus”, “response to stress” and interestingly “embryo development” in cluster 11 was unexpected, since it has been shown, that the anther pre-treatment activates plant defense gene expression in response to mannitol solution and cold stress treatment [31]. Considering the decreasing expression levels with initiated embryogenesis the latter GO term most likely represents suppressors of embryo development.

### sRNA sequencing results

**Fig. 5:**
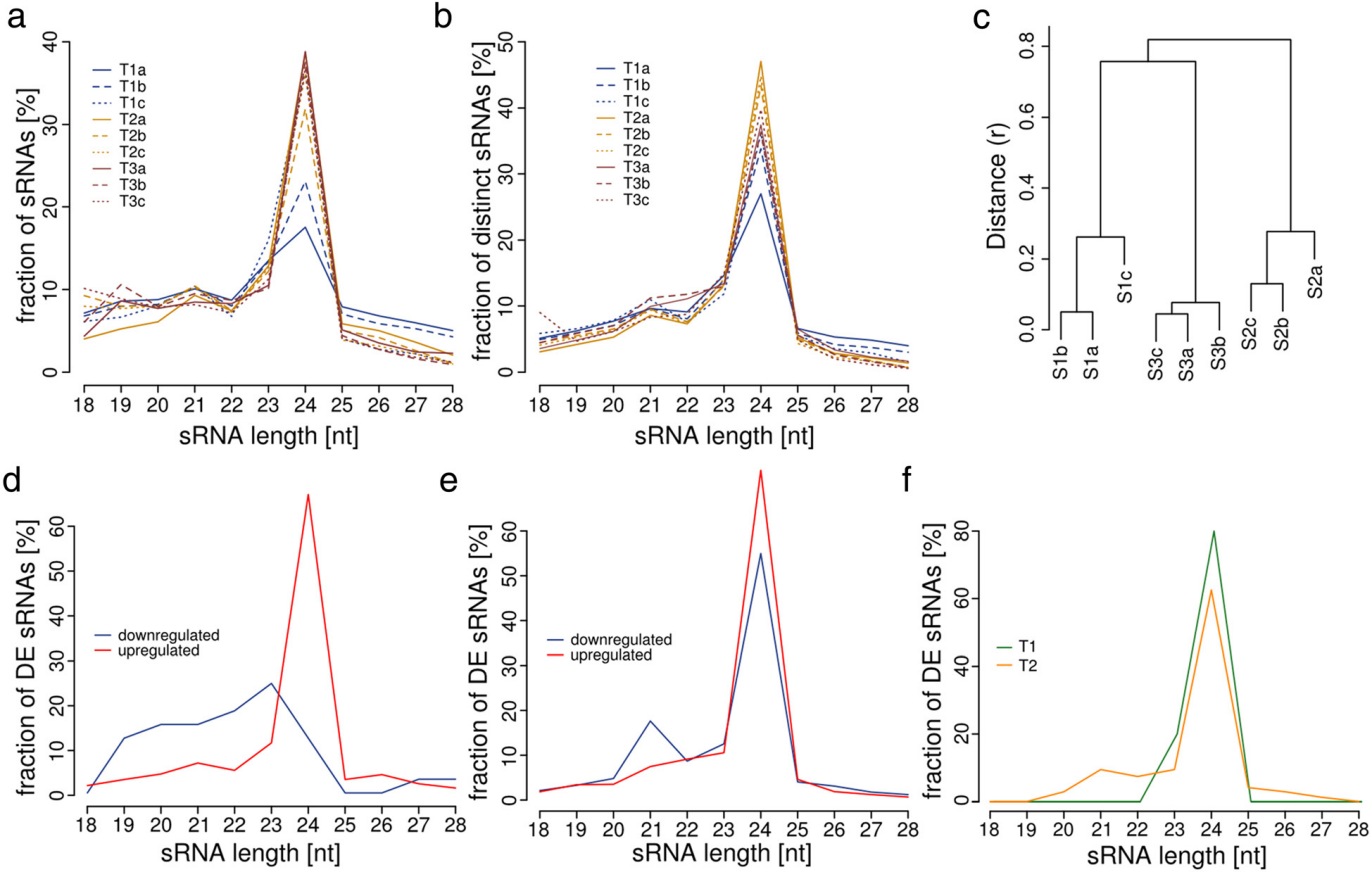
Results from sRNA-seq expression analysis. **a** Total sRNA read length distribution for all replicates, **b** Distinct sRNA read length distribution for all stage replicates, **c** Correlation based clustering analysis for sRNA-seq expression values between the replicates of all microspore stages. **d** Length distribution of DE sRNAs in the first transition (T1 between stages S1 and S2, downregulated n = 37, upregulated n = 830), **e** Length distribution of DE sRNAs in the second transition (T2 between stages S2 and S3, downregulated n = 4,240, upregulated n = 8,868) **f** Length distribution of sRNAs negatively correlated with predicted target transcripts with DE pattern (T1 n = 5, T2 n = 243)

The sRNA-seq resulted in 92.54 Mio. clean reads, with 9.71 Mio. to 10.79 Mio. reads per library (see Table 1). In total 19.63 Mio. distinct sequences were obtained, with 1.8 Mio. to 4 Mio. distinct sequences per library (see Table 1). The sRNA length distribution exhibits a peak at 24-nt for all replicates of all three stages, representing the most abundant short interfering RNAs (siRNA). However, two of the three replicates from S1 showed a smaller fraction of 24-nt sRNAs (Fig. 5a). The length distribution of distinct sRNA reads exhibited an additional peak for 21-nt sRNAs (Fig. 5b), a fraction of which most likely represents microRNAs (miRNA). The fraction of 24-nt sRNAs exhibited a higher variability than given for the total sRNA length distribution in contrast to the sRNA lengths from 15-nt to 20-nt as well as 25-nt to 28-nt, which, except of 20-nt sRNAs, are not representing known functionally active sRNA classes [32].

### sRNA expression analysis

Distinct sRNAs were defined as expressed if their expression was equal or higher than 1 rpmqn, this criterion was satisfied for 63,880 to 70,478 sRNAs per library. A comparison of expression values between all replicates of the three stages revealed a high similarity for the three replicates of each stage (Fig. 5c), again reflecting the overall uniformity of the biological replicates beside the differences in abundance of RNAs of specific length. The variance between replicates for sRNAs is higher than for transcripts. A possible explanation provides the less specific generation of siRNAs from various genomic loci in contrast to the defined gene loci of transcripts. In contrast to the transcript expression, the correlation between the replicates revealed drastic expression changes from S1 to S2 as well as S2 to S3, as the replicates of S2 are less correlated to S1 and S3 than S1 and S3 to each other. This drastic difference to transcript expression pattern might be explainable by stress-induced activation of transposons resulting in the generation of new sets of siRNAs and delayed effects on gene expression by *de novo* methylation of target TEs [33, 34]. Furthermore this difference might be attributed to the different isolation procedures of microspores for mRNA and sRNA sequencing: In contrast to individual selection of microspores with specific morphology for mRNA sequencing (Fig. 1), the gradient centrifugation, we used to isolate microspores for sRNA sequencing, enrich for living microspores only and thus more likely include sRNAs from microspores undergoing cell fates other than embryogenesis.

DE sRNAs were determined from all sRNAs with an expression level of at least 2 rpmqn in the higher expressed stage, a minimum of two-fold expression change. The expression analysis with these thresholds resulted in 867 DE sRNAs for T1, with 830 (95.73 %) being upregulated and 37 (4.27 %) being downregulated (Additional file 10: Table S9, Additional file 11: Table S10). The upregulated sRNAs account primarily to 24-nt sRNAs while the downregulated sRNAs are scattered from 19-nt to 23-nt (Fig. 5d). For T2 13,108 DE sRNAs were identified in total, with 8,868 (67.65 %) being upregulated and 4,240 (32.35 %) being downregulated (Additional file 10: Table S9, Additional file 11: Table S10). In T2, 24-nt sRNAs accounted for the majority of up as well as downregulated sRNAs. Furthermore, the downregulated sRNAs exhibited a high fraction of 21-nt sRNAs (Fig. 5e). 304 of the DE sRNAs overlap between T1 and T2 that all are of 24-nt length.

In the various developmental stages analyzed, 66 of 119 known mature miRNAs of wheat listed in miRBase release 21 [35] were found to be expressed. Three of these (tae-miR9669-5p, tae-miR397-5p, and tae-miR9658-3p) showed upregulation, whereas one (tae-miR9672b) showed downregulation in T2. Consistent with a putative role in microspore embryogenesis, which involve the generation of undifferentiated multicellular structures at first, miR397 was shown to be highly expressed in undifferentiated but not in differentiated rice embryogenic calli from somatic tissues [36]. Interestingly, miR397 is upregulated under cold conditions [37] and overexpression resulted in higher cold stress tolerance in *Arabidopsis* [38]. In wheat microspores, cold inducibility of miR397 might be reduced or delayed, since we revealed no upregulation in S2 right after the cold stress treatment but in the later stage S3. Another miRNA, which might be involved in the regulation of androgenesis, is tae-miR9658, since it was shown to be highly expressed in developing grains but less abundant in vegetative tissues [39].

### Prediction of sRNA target transcripts

To identify potential regulatory effects of sRNAs on mRNAs we predicted sRNA targets among the assembled transcripts for all DE sRNAs. The target prediction resulted in 97 putative target transcripts for DE sRNAs in T1 and 1,179 putative sRNA target transcripts in T2. Five sRNA/target pairings in T1 exhibited DE transcripts and a strong negative correlation between sRNA and target expression. All these targets were downregulated from S1 to S2. For T2, we found 251 DE target transcripts of which 133 exhibit a strong negative correlation with the targeting sRNA expression values, 86 of these targets were up and 47 were downregulated from S2 to S3 (Additional file 12: Table S11). No DE targets could be identified among the assembled contigs for any of the 4 DE known miRNAs listed in miRBase release 21 [35].

Although we used different microspore isolation procedures for mRNA and sRNA sequencing, both datasets are highly related by identical tissue culture conditions and staging. Thus, a negative correlation between sRNA and targeted transcript expression should support the involvement of the sRNA in regulation of the target transcript either by post-transcriptional gene silencing (PTGS) or transcriptional gene silencing (TGS) [40]. The targeted transcripts identified include a number of genes indicated to be involved in embryogenesis or even androgenesis, such as *HDA19* (transcript_2818), as already discussed in the context of the DE transcripts analysis, which is downregulated in T2. *HDA19* has been shown to contribute to the repression of embryogenesis-related genes after germination [27]. It has been furthermore shown that histone deacetylase inhibitors promote totipotency to microspores [41]. Chromatin modifications are suggested to be required for the accessibility of embryogenesis-related genes [9]. Another interesting gene is *PROLIFERA* (*PRL*), represented by transcript_9383, which is upregulated in T2. *PRL* has been shown to be involved in megaspore and embryo development but not in the developing microgametophyte [42] and thus might represent the deviation from the gametophytic cell fate. *TaCer1* (transcript_178), which is highly increased in microspores after stress treatment (S2) and is 528-fold downregulated in T2, represents *ECERIFERUM1* a putative decarbonylase involved in cuticular wax biosynthesis from very long chain fatty acids (VLCFA), which can be regulated by various stresses [43]. It was suggested that *Cer1* is involved in stress tolerance by modulating the aldehydes/alkanes ratio of cuticular wax in wheat as well as other plants [43–46]. A previous study suggested the involvement of wax biosynthesis from VLCFA in wheat microspore embryogenesis as a pathway resulting in signal molecules leading to controlled cell division in absence of surrounding tissue [47]. Overall, only 48 of 666 DE target genes potentially regulated by sRNAs obtained an annotation. We expect the remaining set of transcripts to contain important regulatory genes involved in the alteration of cell fate towards embryogenic development, not yet discovered by other studies.

### Prediction of miRNA precursors

To differentiate between potential PTGS and TGS, miRNA precursors on wheat genomic sequences were predicted. The prediction of miRNA precursors (pre-miRNA) revealed in total 1,717 distinct candidates (Additional file 13: Table S12). 39 pre-miRNAs candidates showed homology to known pre-miRNAs (Table 4). The comparison of the predicted mature miRNAs with the identified sRNA/target pairs revealed two putative miRNAs, namely sRNA 1167446 potentially targeting transcript_1777 with high homology to the *Aegilops tauschii* predicted protein F775_10365 of unknown function and sRNA 6202839 targeting transcript_5689 with high homology to the wheat cDNA TRAES_3AS_647411E39.2 of unknown function. These results with nearly no consensus between predicted mature miRNAs and the sRNAs with predicted targets suggests that miRNA-mediated post-transcriptional regulation does not explain the majority of the predicted sRNA/target pairs with high negative correlation. In the light of the high number of chromatin modifiers identified to be expressed, it is assumed that different sRNA-guided TGS mechanisms such as chromatin modifications and DNA methylation are involved in the induction of microspore embryogenesis in wheat. This assumption is supported by the high variability of the number of distinct 24-nt sRNAs between stages (Fig. 5b), as well as the high fraction of DE 24-nt sRNAs negatively correlated with their potential target transcript expression (Fig. 5f), as 24-nt sRNAs were shown to be predominantly involved in TGS [32].

**Table 4:** Numbers of pre-miRNA candidates with homology to known pre-miRNAs in miRBase release 21 [35]

Predicted pre-miRNA	BLASTn top hit in miRBase release 21	Organism
miRCandidate1467	osa-MIR159e	*Oryza sativa*
miRCandidate1	ata-MIR166a	*Aegilops tauschii*
miRCandidate1079	ata-MIR166b	*Aegilops tauschii*
miRCandidate770	ata-MIR166e	*Aegilops tauschii*
miRCandidate1062	bdi-MIR166g	*Brachipodium distachion*
miRCandidate923	bdi-MIR166g	*Brachipodium distachion*
miRCandidate1425	osa-MIR168a	*Oryza sativa*
miRCandidate437	ata-MIR393	*Aegilops tauschii*
miRCandidate1319	osa-MIR396c	*Oryza sativa*
miRCandidate675	ata-MIR398f	*Aegilops tauschii*
miRCandidate90	tae-MIR1122a	*Triticum aestivum*
miRCandidate1040	tae-MIR1128	*Triticum aestivum*
miRCandidate1068	tae-MIR1128	*Triticum aestivum*
miRCandidate1370	tae-MIR1128	*Triticum aestivum*
miRCandidate30	tae-MIR1128	*Triticum aestivum*
miRCandidate342	tae-MIR1128	*Triticum aestivum*
miRCandidate45	tae-MIR1128	*Triticum aestivum*
miRCandidate941	tae-MIR1128	*Triticum aestivum*
miRCandidate1469	tae-MIR1135	*Triticum aestivum*
miRCandidate1585	tae-MIR1135	*Triticum aestivum*
miRCandidate471	tae-MIR1135	*Triticum aestivum*
miRCandidate720	tae-MIR1135	*Triticum aestivum*
miRCandidate1023	tae-MIR1136	*Triticum aestivum*
miRCandidate1678	tae-MIR1136	*Triticum aestivum*
miRCandidate1641	tae-MIR5048	*Triticum aestivum*
miRCandidate1033	tae-MIR5084	*Triticum aestivum*
miRCandidate1332	tae-MIR5084	*Triticum aestivum*
miRCandidate171	tae-MIR9653a	*Triticum aestivum*
miRCandidate166	tae-MIR9653b	*Triticum aestivum*
miRCandidate177	tae-MIR9653b	*Triticum aestivum*
miRCandidate823	tae-MIR9657c	*Triticum aestivum*
miRCandidate689	tae-MIR9661	*Triticum aestivum*
miRCandidate309	tae-MIR9671	*Triticum aestivum*
miRCandidate1069	tae-MIR9672a	*Triticum aestivum*
miRCandidate1146	ata-MIR9674a	*Aegilops tauschii*
miRCandidate883, miRCandidate892	tae-MIR9674a	*Triticum aestivum*
miRCandidate930	ata-MIR9674a	*Aegilops tauschii*
miRCandidate884	tae-MIR9674b	*Triticum aestivum*
miRCandidate1355	tae-MIR9777	*Triticum aestivum*

## Conclusions

Our study provides the first large-scale transcriptome dataset for microspore embryogenesis induction in wheat. The *de novo* assembly and mapping to public wheat cDNA sequences resulted in a high number of novel transcripts. A major part of these might be presumably largely specific transcripts for microspore embryogenesis induction. A GO annotation revealed a large fraction of stress as well as embryo development related transcripts. Many transcripts were found to be specifically expressed in microspores undergoing their first visible nuclear division, numerous with functions in embryogenesis or epigenetic mechanisms. While the inductive treatment resulted in 756 DE transcripts, the following transition resulting in first nuclear divisions exhibits a larger set of 5,269 DE transcripts. A GO enrichment analysis for the DE transcripts revealed metabolism and biosynthesis related transcripts to be downregulated in both stage transitions whereas chromatin related transcripts were found to be enriched and thus represent a dedifferentiation of the developing microspore followed by a reprogramming towards embryogenic development. The sRNA sequencing mirrors the transcriptome in terms of numbers with 867 DE sRNAs in the first and 13,108 DE sRNAs in the second stage transition. Prediction of sRNA targets identified a large number of putative target transcripts, which contained genes previously shown to be involved in microspore or zygotic embryogenesis. Our results suggest epigenetic mechanisms related to TGS rather than miRNA-based PTGS to be largely involved in the reprogramming of the microspore developmental fate. These results and the generated sequencing resources will contribute to a deeper understanding of the molecular mechanisms involved in the induction of wheat microspore embryogenesis.

## Methods

### Plant material

The Bulgarian winter wheat cultivar ”Svilena”, European Wheat Database accession: BG 2001-TRT-AE-125 [48], which is highly responsive to stress-induced microspore embryogenesis [12] was used for this study. Seeds of this cultivar were initially provided to us by the Institute of Wheat and Sunflower "Dobrudja", General Toshevo, Bulgaria and propagated by us thereafter. Donor plants were grown as described in Rubtsova et al. 2013 [13]. The inductive pre-treatment of spikes for 10 days and the following in vitro culture of anthers was performed as described in Rubtsova et al. 2013 [13].

### Microspore isolation

Two different methods were used for microspore isolations: a) manual sampling for small sample sizes and b) purification of microspores using gradient centrifugation for bigger sample sizes. Three stages of microspore embryogenesis were isolated by both methods.

For transcriptome analysis, microspores were manually sampled under sterile conditions using a 10x objective (100x magnification) and an Eppendorf CellTram vario (Eppendorf AG, Hamburg, Germany). The first stage (S1) represented untreated microspores at the late vacuolated uninucleate stage and were isolated from freshly harvested spikes. At the second time point (S2), slightly enlarged microspores with a star-like structure were sampled directly after a 10-days cold pre-treatment of the spikes. For the third stage (S3), enlarged microspores showing first visible karyokinesis were isolated from in-vitro anther culture. Karyokinesis was generally visible after 4-8 days in culture. During this time, the developmental stages of the microspores were checked daily. When 5-10 % of microspores showed visible karyokineses, all anthers of one petri dish were used for isolation. Extensive preliminary tests were performed, which yielded in the isolation of only ten microspores from 2-8 anthers at a time to keep isolation time short. Microspores were isolated in 0.4 M mannitol, directly frozen in 10 μl 0.4 M mannitol with liquid nitrogen, and stored at -70 °C until further processing.

For sRNA analysis, the same in vitro culture conditions were applied and a gradient isolation of microspores was performed, which enriched for viable microspores but do not select for specific morphology. Also here, extensive preliminary tests were performed to optimize the concentration and purity of the individual developmental microspore stages S1 – S3. Since the amount of responding microspores is reduced with each stage, the number of spikes had to be adapted, e.g. for S3 up to 24 spikes were used for one isolation. For optimized microspore isolation, anthers were collected in 0.4 M mannitol, homogenized, sieved and centrifuged at 98 g for 5 min to pellet the microspores. The precipitate was concentrated and washed 2 times in 0.4 M mannitol at 98 g for 5 min. The resuspension was stacked on 20 % maltose and centrifuged at 98 g for 4 min. During this step, all cell debris and non-viable microspores were discarded. The interphase was washed with 0.4 M mannitol and centrifuged at 98 g for 10 min before resuspending in a small amount of 0.4 M mannitol. The purity of each isolation was checked by light microscopy and the concentration of microspores was determined by counting microspores in 1 μl suspension in triplicate.

### RNA preparation and sequencing

For RNA-seq and sRNA-seq, 3 biological replicates for each of the 3 stages were used. For RNA-seq, mRNA was isolated from 100 hand-sorted microspores and cDNA libraries were generated according to the protocol described in Lê *et al.* 2005 [49]. RNA-seq library preparation of the sample replicates indexed with unique nucleic acid identifiers was performed using Illumina Nextera DNA Library Kit (Illumina Inc, San Diego, CA, USA). For sRNA-seq, total RNA was isolated using the mirVana miRNA Isolation Kit (Life Technologies Corp., Carlsbad, CA, USA) from ~100,000 gradient-sorted microspores. The quality of the RNA samples was tested and verified by BGI Tech Solutions Co., Ltd. (Hong Kong) on a 2100 Bioanalyzer (Agilent, Santa Clara, CA, USA) prior to sequencing library generation with the TrueSeq Small RNA Library Preparation Kit. Illumina sequencing of 50 bp single-end reads was performed on Illumina Hi-Seq 2000 instruments (Illumina Inc, San Diego, CA, USA) by BGI Tech Solutions Co., Ltd. (Hong Kong).

### Sequencing data processing

The RNA-seq data was generated with CASAVA 1.8.1 (Illumina Inc, San Diego, CA, USA) and initially processed by BGI Tech Solutions Co., Ltd. (Hong Kong) to remove adapter sequences, contamination and low-quality reads. The preprocessed RNA-seq reads were quality-trimmed to 99.9 % sequencing quality using a custom Java program using the Picard API [50]. Trimmed reads with less than 40 bp were discarded. The sRNA-seq reads were trimmed from adapter sequences and quality-trimmed to 99.9 % sequencing quality. All sRNA reads longer than 15 nt were retained for further analysis.

### Transcriptome *de novo* assembly and annotation

All trimmed RNA-seq reads from all replicates of all three stages were merged and used for *de novo* assembly of a wheat microspore embryogenesis transcriptome. The assembly was performed using Trinity version r2013-08-14 [16] with default parameters.

The contigs were aligned to the NCBI non-redundant protein (nr) database (downloaded 12/13/2013, ftp.ncbi.nlm.nih.gov/blast/db) using BLASTx (version 2.2.29+) [51] with maximum e-value of 10^-10^ and a limitation to the 20 most significant alignments. Based on this mapping the contigs were GO annotated using Blast2GO [18] and Trinotate [19].

The assembled contigs were mapped to wheat cDNA sequences from ensembl release 26 [20] using BLASTn (version 2.2.29+) [51] to identify fragmented assemblies. Multiple contigs mapping to one cDNA were clustered to a single transcript identifier. GO terms obtained for transcripts with fragmented assembly were merged.

### sRNA annotation

The sRNA sequences were mapped to mature miRNAs listed for wheat in miRBase release 21 [35] using the sequence aligner Bowtie (version 1.01) [52] without mismatches.

### Expression analysis

The expression patterns of the transcripts identified by the *de novo* assembly were calculated by counting all reads uniquely mapping to the contigs of transcript clusters using the sequence aligner Bowtie (version 1.01) [52] allowing one mismatch. The sample replicates were hierarchically clustered based on their Pearson correlation coefficients.

The sRNA sequences were collapsed to unique sequence reads and read counts were determined.

Read count data from transcriptome as well as sRNA were individually normalized by quantiles normalization [53] with a modification preventing the allocation of expression values to transcripts not expressed in the sample. The quantile normalized expression values were finally scaled to 1 Mio. quantiles normalized reads (rpmqn). The sample replicates were hierarchically clustered based on their Pearson correlation coefficients with the complete linkage method in R [54].

### Differential expression analysis

The DE of transcripts and analogously of sRNAs was tested individually for both transitions (T1 from stage S1 to S2, T2 from S2 to S3) for all transcripts or sRNAs, respectively, with at least two-fold expression change between the stages a minimal expression of 1 rpmqn in the higher expressed stage and a maximal standard deviation for the stage replicates of 25 % from the average expression of the stage. The significance of DE was tested by Student's t-tests with a FDR of 5 % [55] in a custom Java program using the Java Statistical Classes API [56].

### Cluster analysis of differentially expressed transcripts

A grouping of the replicates of all microspore stages was performed by principal component analysis in R [54] based on z-normalized expression data for all transcripts as well as separately for all DE transcripts in at least one transition.

A k-means cluster analysis of mean z-normalized stage replicate transcript expression means with k set to 12 clusters was performed in Gene Cluster 3.0 using the centered correlation as dissimilarity measure [57].

### GO enrichment analysis for transcript expression pattern clusters

The enrichment for GO terms was tested for all clusters in a bootstrap analysis with 1 Mio. runs in a custom Java program, all terms with at least 5 transcripts and a FDR [55] below 5 % were considered to be enriched.

### sRNA target prediction

A sRNA target prediction was performed for all DE sRNAs on the *de novo* assembled contigs from the RNA-seq using psRNATarget with default parameters [58]. All putative sRNA/target pairs were subsequently filtered by correlation analysis of the sRNA and transcript expression. Only sRNA/target pairs with strong negative correlation were retained as putatively sRNA regulated transcripts.

### miRNA prediction

A prediction of miRNA precursors for the identification of miRNAs was performed using miRDeep-P (version 1.3) [59] individually for all stage replicates using the IWGSP1 chromosome based draft sequence [60] as genome. The pre-miRNAs were searched for homology to known pre-miRNAs in miRBase release 21 [35] using BLASTn (version 2.2.29+) [51]. The predicted mature miRNAs were filtered for sRNAs with predicted targets from the sRNA target prediction.

### Ethics approval and consent to participate

Our experimental research complied with institutional, national, or international guidelines. We did not use any endangered species and complied with the Convention on the Trade in Endangered Species of Wild Fauna and Flora.

### Consent to publish

Not applicable

### Availability of data and materials

The data sets supporting the results of this article are available in the NCBI BioProject repository, under accession PRJNA297977, [http://www.ncbi.nlm.nih.gov/bioproject/PRJNA297977]. This Transcriptome Shotgun Assembly project has been deposited at DDBJ/ENA/GenBank under the accession GDTJ00000000. The version described in this paper is the first version, GDTJ01000000.

## Additional files

Additional file 1: Table S1. Transcript reconstruction. Reconstruction of *de novo* assembled contigs to wheat transcripts based on known wheat cDNAs. (XLS 1968 kb) Additional file 2: Table S2. BLASTx hits of transcripts. BLASTx top hit results for *de novo* assembled contigs of restructured transcripts. (XLS 4120 kb) Additional file 3: Table S3. GO annotation for transcript reconstruction. GO annotation for restructured *de novo* assembled transcripts based on functional homology using Blast2GO [16] and Trinotate [17]. (XLS 1212 kb) Additional file 4: Table S4. GO annotation counts for the assembled transcripts. Summary of retrieved GO annotations for *de novo* assembled, restructured transcripts. (XLS 263 kb) Additional file 5: Table S5. Stage specific transcripts. Listing of restructured *de novo* assembled transcripts with stage-specific expression (at least 0.5 qnrpm in all replicates) including BLASTx top hits. (XLS 88 kb) Additional file 6: Table S6. DE transcripts in T1 and T2. Listing of transcript identifiers for DE, restructured *de novo* transcripts up- and downregulated in the stage transitions. (XLS 275 kb) Additional file 7: Figure S1. PCA results for DE transcripts. PCA plot for DE transcript expression patterns resulting in clear separation of sample stage replicates. (PNG 55 kb) Additional file 8: Table S7. Enriched GO terms for DE transcripts in the stage transitions. Listing of GO terms enriched for DE transcripts in stage transitions seperately for up- and downregulated expression patterns with term description, number of transcripts, and p-value. (XLS 35 kb) Additional file 9: Table S8. Enriched GO terms for DE transcripts clustered by expression patterns. Listing of GO terms enriched for k-means clustered expression patterns of DE transcripts with description, number transcripts, and p-value. (XLS 27 kb) Additional file 10: Table S9. DE sRNAs in T1 and T2. Listing of identifiers of DE sRNA up- and downregulated in the stage transitions. (XLS 349 kb) Additional file 11: Table S10. Sequences of DE sRNAs and predicted miRNAs. Listing of all DE sRNAs as well as miRNAs with their sRNA identifier (XLS 1022 kb) Additional file 12: Table S11. Putative sRNA/target pairs identified from negatively correlated DE sRNAs and DE transcripts. Listing of sRNA/target pairs based on negative correlation of DE sRNA and DE transcript expression patterns that were identified using psRNATarget [58]. Stage transitions of DE, correlation coefficient, and transcript BLASTx top hits are listed. (XLS 36 kb) Additional file 13: Table S12. miRNA prediction results. Results from miRNA precursor predictions using miRDeep-P [59] performed from sRNA sequencing data individually for all stage replicates. (XLS 790 kb)

## Abbreviations

cDNA: complementary RNA
DE: differentially expressed
FDR: false discovery rate
GO: gene ontology
mRNA: messenger RNA
miRNA: microRNA
NCBI: National Center for Biotechnology Information
PCA: principal component analysis
pre-miRNA: miRNA precursor
PTGS: post-transcriptional gene silencing
RNA-seq: transcriptome sequencing
rpmqn: reads per million quantile normalized reads
sRNA: small RNA
sRNA-seq: small RNA sequencing
siRNA: small interfering RNA
TGS: transcriptional gene silencing
VLCFA: very long chain fatty acids
