# Supplementary figures and images for: Analysis of wheat microspore embryogenesis induction by transcriptome and small RNA sequencing using the highly responsive cultivar “Svilena”
Source: BMC Plant Biol. 2016 Apr 21;16:97. doi: 10.1186/s12870-016-0782-8 (PMC4839079; doi:10.1186/s12870-016-0782-8)

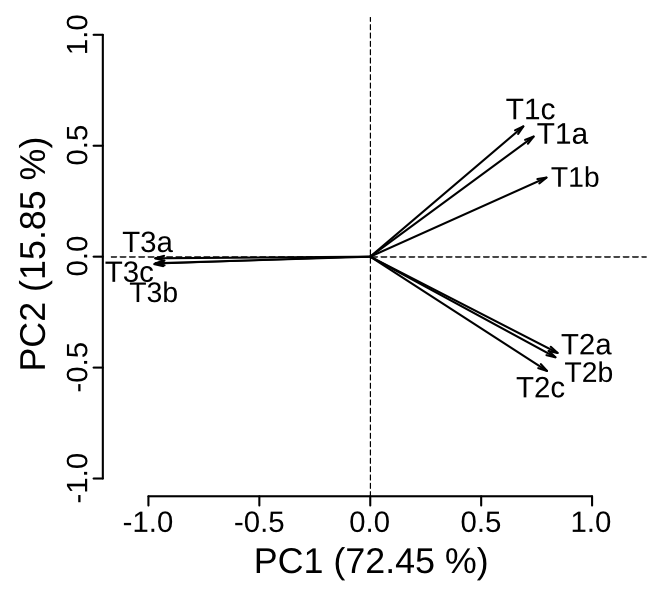

Supplement: Additional file 7: Figure S1. — PCA results for DE transcripts. PCA plot for DE transcript expression patterns resulting in clear separation of sample stage replicates. (PNG 55 kb) [file 12870_2016_782_MOESM7_ESM.png]
